# Operative Treatment of Ascites in Cirrhosis of the Liver

**Published:** 1909-08

**Authors:** 


Operative Treatment of Ascites in Cirrhosis of the Liver. —Dr. N. F. Bogojawlensky (*Zentralb. f. Chir.*, No. 9, 1909) believes that abdominal section itself is responsible for as large a share of the beneficial effect as suture of the omentum in Talma's operation. In the ten cases in which he has operated he has paid special attention to as complete removal of the ascitic fluid as possible.—*International Journal of Surgery.*

---

To relieve entero-colitis or cholera infantum, cleanse intestinal tract with calomel and saline or castor oil; prescribe an easily digested, non-irritating diet. Instead of using opiates, relieve muscular rigidity and pain by using antiphlogistine as hot as can be borne over entire abdominal wall.—*Virgina Medical Semi-Monthly.*

